# *Drosophila* *l(2)23CDe ^A8-1^* and *l(2)23CDf ^A18-2^* are missense alleles of the *SerRS* gene

**DOI:** 10.17912/micropub.biology.002357

**Published:** 2026-09-16

**Authors:** Benjamin Galligos, Giovanna Caime, Alexis Michalski, Jamie Dyer

**Affiliations:** 1 Biology, Rockhurst University, Kansas City, MO, United States; 2 Saint Louis University School of Medicine, St. Louis, MO, United States; 3 University of Missouri-Kansas City School of Dentistry, Kansas City, MO, United States; 4 University of Kansas, Lawrence, KS, United States

## Abstract

Viability requires that certain genes are expressed at the correct levels and at specific times during development. Many genes have already been determined as essential to development, but many more may exist. Numerous lethal mutations have been isolated in *Drosophila melanogaster* that have yet to be identified at the gene level. Here we provide deficiency mapping, complementation testing, and DNA sequencing that suggests the unknown lethal mutations *l(2)23CDe ^A8-1^* and *l(2)23CDf ^A18-2^* are both missense mutations in the *SerRS* gene, which encodes an aminoacyl tRNA synthetase that is required for protein translation.

**Figure 1. Complementation testing of unknown lethal mutations l(2)23CDe A8-1 and l(2)23CDf A18-2 with known mutations in 19 genes located with Df(2L)JS17:** Results suggest that unknown lethal mutations *l(2)23CDe ^A8-1^* and *l(2)23CDf ^A18-2^* do not complement with *SerRS ^KG03126^ .
*

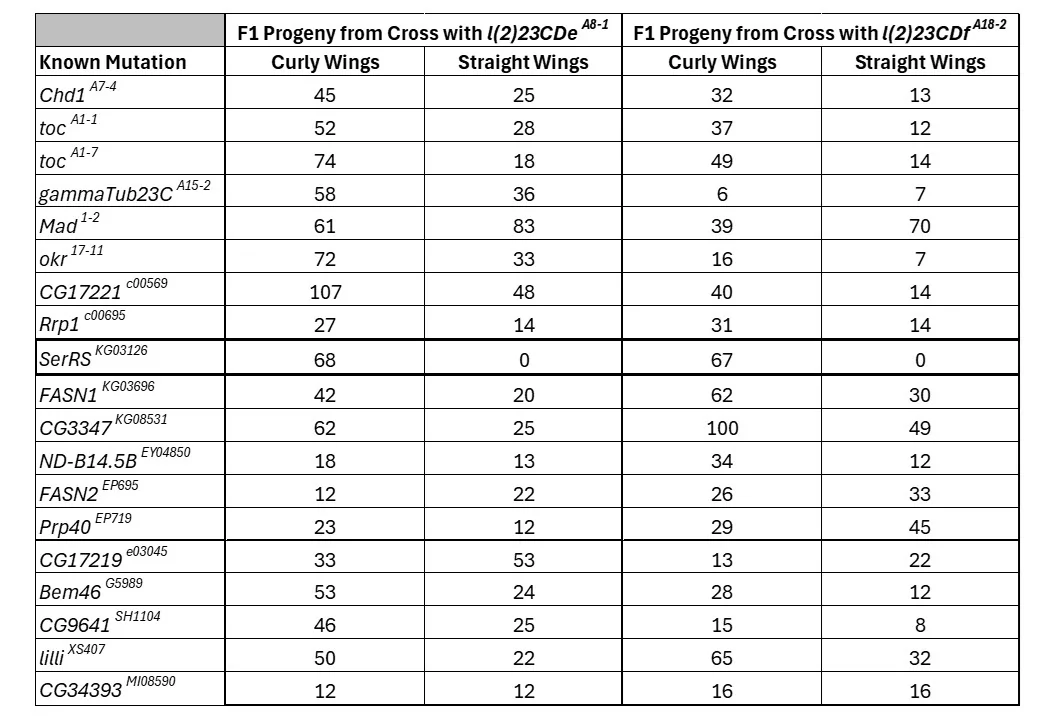



## Description

Numerous genes have been previously determined to be essential for the development of *Drosophila melanogaster* (Wieschaus and Nüsslein-Volhard 2016). Mutations in these genes can result in premature death or failure to develop and therefore, are referred to as lethal mutations. Though many genes are known to be required for viability, there are also over 1,500 previously isolated lethal mutations in *Drosophila* yet to be determined at the gene level (personal communication with the Stock Center). Previous studies have found that about 75% of human genes known to be involved in human disease formation have functional homologs that can be studied in *D. melanogaster* (Verheyen, 2022). Thus, determining which genes are important for development and viability in *Drosophila* may also result in a better understanding of human development and health.

In order to identify the genes mutated in these unknown lethal mutant stocks, we utilized deficiency mapping and complementation testing. With the development of next generation sequencing, the identification of these unknown mutations would seem to be straightforward. However, initial sequencing results of a few stocks identified a large number of variations from the reference genome, making deficiency mapping and complementation testing the more straightforward approach. Deficiency mapping can be used to narrow down the location of an unknown mutation to a region within or outside the deficiency utilized (Hales et al., 2015). Through the use of balancer chromosomes with the *Duox ^Cy^* marker, examining the progeny from deficiency mapping crosses involved determining whether these progeny without the balancer (and therefore, without the *Duox ^Cy^* marker) were viable. If the only surviving F _1_ progeny had curly wings, this would suggest that the unknown lethal mutation was within the deficiency.

Deficiency mapping using *Df(2L)JS17* determined that unknown lethal mutations *l(2)23CDe ^A8-1^* and *l(2)23CDf ^A18-2^* were likely located within the deficiency located between bands 23C1 and 23E2 on chromosome 2 in *Drosophila melanogaster* (Öztürk-Çolak, et al., 2024). The results of these deficiency crosses found 52 of 56 F _1_ progeny displayed curly wings for *l(2)23CDe ^A8-1^* and all 38 F _1_ progeny displayed curly wings for *l(2)23CDf ^A18-2^* . These results were then verified with a second round of crosses.

In order to determine the location of these two unknown lethal mutations at the gene level, complementation testing was utilized. When beginning this project, 19 different loss of function mutant stocks in genes mapped within the *Df(2L)JS17* deficiency were present at the Bloomington *Drosophila* Stock Center at Indiana University Bloomington (Cook et al., 2010; Öztürk-Çolak, et al., 2024). Complementation testing was performed with these stocks and the unknown lethal mutation stocks that were determined to be within *Df(2L)JS17* . If the resulting F _1_ progeny did not display any flies with straight wings, this would suggest that the two mutations did not complement and therefore, the unknown mutation is likely within the same gene as the known mutation. Results from these crosses suggested that both *l(2)23CDe ^A8-1^* and *l(2)23CDf ^A18-2^* did not complement with *SerRS ^KG03126^* (Table 1). These results were verified with a second set of complementation test crosses. Taken together, these results suggest that both *l(2)23CDe ^A8-1^* and *l(2)23CDf ^A18-2^* are mutations within the *SerRS* gene, which is involved in protein translation (Arsham and Neufeld 2009).

In order to confirm our results, the *SerRS* gene was amplified via PCR from the stocks containing *l(2)23CDe ^A8-1^* and *l(2)23CDf ^A18-2^* mutations and then sequenced to identify any lesions in these genes. Lethal mutation *l(2)23CDe ^A8-1^* was determined to have a missense mutation in the *SerRS* coding region, which would result in a change of an arginine to cysteine at amino acid 318 in the SerRS protein. Lethal mutation *l(2)23CDf ^A18-2^* was also determined to have a missense mutation in the *SerRS* coding region, resulting in a change of a glutamine to leucine at amino acid 327 in the SerRS protein. As previous studies have determined that SerRS serves a canonical role in protein translation as well as non-canonical roles in processes such as neuronal necrosis and lysosomal regulation, mutations that result in changes to the amino acid sequence of this protein affect fundamental cellular processes and therefore results in lethality (Arsham and Neufeld 2009; Herzog et al., 2009; Lei et al., 2017).

Due to the similarities between genes found in humans and *Drosophila* , the genes needed for viability in *Drosophila* may also be vital for human development. The increased knowledge and findings stemming from this project may provide a better understanding of the genetic requirements for viability and development in humans.

## Methods

*Drosophila melanogaster* stocks were obtained from the Bloomington *Drosophila* Stock Center. Specific stocks used in these experiments are included in Table 2. Flies were maintained at room temperature in vials and bottles with Nutri-Fly® BF fly food (Genesee Scientific). Flies were examined using stereo microscopes and CO _2_ pads for anesthetizing.

Deficiency mapping was performed by crossing virgin females and males from the deficiency and unknown lethal mutation stocks. The resulting F _1_ progeny were scored for curly or straight wings to determine the location of the unknown mutation with respect to the deficiency.

Complementation testing was performed by crossing virgin females and males from the unknown lethal mutation stocks to stocks with known mutations in genes located within the deficiency. The resulting F _1_ progeny were examined for curly or straight wings to determine whether the unknown lethal mutations did not complement the known mutations in genes within the deficiency.

Genomic DNA was obtained from *Drosophila* stocks using the Qiagen DNeasy Blood & Tissue Kit, following the manufacturer's protocol. The *SerRS* gene was amplified from 2.5µl of genomic DNA from the mutant stocks and wild type flies using 2X Phusion Master Mix according to the manufacturer's protocol for 40 cycles. Primers used to amplify the *SerRS* gene were SerRS-F 5´- CCACCATCGCTTTCCAGCA - 3´ and SerRS-R 5´- GCACAGCTTGATCCAGTTTAA - 3´. PCR samples were purified using Qiagen QIAquick Gel Extraction Kit according to the manufacturer's protocol. The resulting PCR bands were sent for DNA sequencing at the University of Missouri Genomics Technology Core for each fly stock. Results were analyzed using the freely available A plasmid Editor (ApE) application and FinchTV to identify the nature of the mutations.

## Reagents

**Table:** 

BDSC #	Genotype
1567	* Df(2L)JS17, dpp ^d-ho^ /CyO, P{ry ^+t7.2^ =en1}wg ^en11^*
5722	* l(2)23CDe ^A8-1^ cn ^1^ bw ^1^ /CyO *
5727	*Chd1 ^A7-4^ cn ^1^ bw ^1^ /CyO *
5737	*toc ^A1-1^ cn ^1^ bw ^1^ /CyO *
7036	* l(2)23CDf ^A18-2^ cn ^1^ bw ^1^ /CyO *
7039	*toc ^A1-7^ cn ^1^ bw ^1^ /CyO *
7042	*gammaTub23C ^A15-2^ cn ^1^ bw ^1^ /CyO *
7323	w ^*^ ; Mad ^1-2^ P{ry ^+t7.2^ =neoFRT}40A/CyO
8524	*okr ^17-11^ cn ^1^ bw ^1^ /CyO *
10187	*w ^1118^ ; PBac{w ^+mC^ =PB}CG17221 ^c00569^ /CyO *
10213	*w ^1118^ ; PBac{w ^+mC^ =PB}Rrp1 ^c00695^ /CyO *
12894	*y ^1^ ; P{y ^+mDint2^ w ^BR.E.BR^ =SUPor-P} SerRS ^KG03126^ /CyO; ry ^506^*
13255	*w ^1118^ ; P{y ^+mDint2^ w ^BR.E.BR^ =SUPor-P}FASN1 ^KG03696^ /CyO, P{ry ^+t7.2^ =sevRas1.V12}FK1 *
14733	*y ^1^ ; P{y ^+mDint2^ w ^BR.E.BR^ =SUPor-P}CG3347 ^KG08531^ /CyO; ry ^506^*
15944	*y ^1^ w ^67c23^ ; P{y ^+mDint2^ w ^+mC^ =EPgy2}ND-B14.5B ^EY04850^ /CyO *
17192	*w ^1118^ ; P{w ^+mC^ =EP}FASN2 ^EP695^ /CyO *
17291	*w ^1118^ ; P{w ^+mC^ =EP}Prp40 ^EP719^ /CyO *
18110	*w ^1118^ ; PBac{w ^+mC^ =RB}CG17219 ^e03045^ /CyO *
28472	y ^1^ w ^*^ ; P{w ^+mC^ =EP}Bem46 ^G5989^ /CyO
29489	w ^*^ ; P{w ^+mC^ =lacW}CG9641 ^SH1104^ P{ry ^+t7.2^ =neoFRT}40A, l(2)SH1104 ^SH1104^ /CyO
30716	w ^*^ ; lilli ^XS407^ /CyO, P{w ^+mC^ =GMR-sina.N}2
44796	y ^1^ w ^*^ ; Mi{y ^+mDint2^ =MIC}CG34393 ^MI08590^ /SM6a

## References

[R1] Arsham Andrew M., Neufeld Thomas P. (2009). A Genetic Screen in Drosophila Reveals Novel Cytoprotective Functions of the Autophagy-Lysosome Pathway. PLoS ONE.

[R2] Cook Kevin R., Parks Annette L., Jacobus Luke M., Kaufman Thomas C., Matthews Kathy (2010). New research resources at the Bloomington Drosophila Stock Center. Fly.

[R3] Hales Karen G, Korey Christopher A, Larracuente Amanda M, Roberts David M (2015). Genetics on the Fly: A Primer on the
*Drosophila*
Model System. Genetics.

[R4] Herzog Wiebke, Müller Katja, Huisken Jan, Stainier Didier Y.R. (2009). Genetic Evidence for a Noncanonical Function of Seryl-tRNA Synthetase in Vascular Development. Circulation Research.

[R5] Lei Ye, Liu Kai, Hou Lin, Ding Lianggong, Li Yuhong, Liu Lei (2017). Small chaperons and autophagy protected neurons from necrotic cell death. Scientific Reports.

[R6] Öztürk-Çolak Arzu, Marygold Steven J, Antonazzo Giulia, Attrill Helen, Goutte-Gattat Damien, Jenkins Victoria K, Matthews Beverley B, Millburn Gillian, dos Santos Gilberto, Tabone Christopher J, Perrimon Norbert, Gelbart Susan Russo, Broll Kris, Crosby Madeline, dos Santos Gilberto, Falls Kathleen, Gramates L Sian, Jenkins Victoria K, Longden Ian, Matthews Beverley B, Seme Jolene, Tabone Christopher J, Zhou Pinglei, Zytkovicz Mark, Brown Nick, Antonazzo Giulia, Attrill Helen, Goutte-Gattat Damien, Larkin Aoife, Marygold Steven, McLachlan Alex, Millburn Gillian, Pilgrim Clare, Öztürk-Çolak Arzu, Kaufman Thomas, Calvi Brian, Campbell Seth, Goodman Josh, Strelets Victor, Thurmond Jim, Cripps Richard, Lovato TyAnna, FlyBase Consortium (2024). FlyBase: updates to the
*Drosophila*
genes and genomes database. GENETICS.

[R7] Wieschaus Eric, Nüsslein-Volhard Christiane (2016). The Heidelberg Screen for Pattern Mutants of
*Drosophila*
: A Personal Account. Annual Review of Cell and Developmental Biology.

